# Visualization of Respiratory Commensal Bacteria in Context of Their Natural Host Environment

**DOI:** 10.3389/fmicb.2021.678389

**Published:** 2021-06-04

**Authors:** Joao P. P. Bonifacio, Mirco Schmolke

**Affiliations:** Microbiology and Molecular Medicine Department, University of Geneva, Geneva, Switzerland

**Keywords:** *L. murinus*, lung tissue, microbiome, commensal bacteria, *in situ* hybridization, RNAScope

## Abstract

Commensal microbes are an integral component of mammalian physiology. 16S rRNA gene-specific next generation sequencing from DNA of total organs, swabs or lavages has revolutionized the characterization of bacterial communities in virtually every ecological niche of the body. Culturomics, next allowed the isolation and characterization of commensal bacteria in the lab and the establishment of artificial communities of bacteria, which were eventually reintroduced in model organisms. Spatial organization of microbiota within a given host environment is critical to the physiological or pathological phenotypes provoked by commensal microbiota. *In situ* hybridization (ISH) is a complementary technique to sequencing and culturing to visualize the presence of individual bacterial operational taxonomic unit (OTUs) in context of the colonized organ. We recently applied highly sensitive *in situ* RNA hybridization to detection of commensal bacteria in low abundance respiratory tract samples of mice housed under specific pathogen free conditions. This technique allows species-specific detection of living bacteria using RNAScope^TM^ technology, while preserving the natural environment of the organ. We here provide a detailed step-by-step protocol describing the detection of commensal lung bacteria in respiratory tissue.

## Introduction

Characterization of bacterial communities in various ecological niches of human or animal bodies largely relies today on 16S rRNA gene specific next generation sequencing. This highly sensitive and valuable technique allows quasi unbiased quantification and identification of bacteria from DNA of swabs, lavages or total tissue samples (Human Microbiome Project Consortium, 2012). It does, however, not distinguish between living and dead bacteria and resolution of spatial organization of the identified bacteria within a given niche is limited to the choice of organ section used for DNA isolation. Complementary analysis of shotgun DNA sequencing allows deeper insight into the physiological state of a given group of living bacteria under changing environmental conditions (Westermann et al., 2016; Griesenauer et al., 2019; Becattini et al., 2021) since bacterial RNA is rather short-lived. Culturomics, that is the isolation and amplification of bacteria by using multiple growth conditions, allows further detailed characterization of bacterial species which were previously considered unculturable (Sibley et al., 2011; Browne et al., 2016). As a complement to these techniques we present here a protocol for RNA-based *in situ* hybridization to detect commensal or pathogenic bacteria in a sensitive fashion in context of the host organ. Previous studies have used fluorescent based approaches to determine spatial organization of commensal microbiota in the intestine and lung (Yun et al., 2014; Tropini et al., 2017; Welch et al., 2017) using phylum specific probes. We extended this approach recently to the respiratory tract of mice, which displays 10^4^ to 10^5^-fold less bacterial content than the intestine (Yildiz et al., 2020). This technique allowed us to identify tissue associated bacteria in the large airways of the lung, with probes detecting all eubacteria or only specific species. We here provide a detailed step-by-step protocol describing the detection of commensal lung bacteria in respiratory tissue, which could easily be adapted to other tissues or other host species.

## Materials and Equipment

### Animals

Animals should be housed and treated according the respective national animal welfare guidelines. Hygiene standards of the respective animal facility will largely contribute to the colonization of the investigated animals (Laukens et al., 2015; Rausch et al., 2016). We base this protocol on specific pathogen free housed mice, but we see no limitations on extending it to conventionally housed or wild animals.

### Extraction and Fixation of Mouse Lung Tissue

Scissors (F.S.I.), micro-dissecting forceps (F.S.I.) Falcon 15-mL conical centrifuge polypropylene tubes (Thermo Fisher Scientific), Paraformaldehyde 4% (Merck), Ethanol 70%.

### Paraffinization and Histological Cuts

Cooling block grid (Leica), histology cassettes (Leica) microscope slides (Thermo Fisher Scientific), microtome (Leica RM2235), Paraplast (Leica), Ethanol with 2% methyl ethyl ketone (MEK) (Biosystems), Histo-Sav (Mallinckrodt Baker).

### Deparafinization

Histological grade xylene, ethanol 99.9 grade, Fume-hood, x8 50-mL beakers, dry oven.

### RNAscope—ISH

RNA Scope kit (ADC) containing: RNAScope Hydrogen Peroxide, RNAScope 10X Target Retrieval, RNAScope 50X Wash Buffer, RNAScope Protease Plus, RNAScope Hydrophobic pen, RNAScope probes (#475131 or #451961), RNAScope AMP1-6 Reagents, RNAScope Fast Red-A, RNAScope Fast Red-B, RNAScope EcoMount. Lysozyme (40 μg/mL), 5X SSC Buffer (optional), Meyer’s Solutions (Sigma), Ammonia, Histological grade xylene, Steam Cooker (e.g., Tefal Vitacuisine Compact VS4003 digitale steam cooker Art. #IP095536), Coverslips, Dry Oven, HybEZ Oven, Slide’s Rack for washing.

### Visualization

Brightfield Olympus BX61VS120 with a motorized stage (Olympus LifeSciences) equipped with Plan Apo N 2×/0.08 and U Plan S Apo 100×/1.4 Oil objectives and a Pike F505C VC50 detector (Allied Vision Technology).

## Methods

**FIGURE 1:**
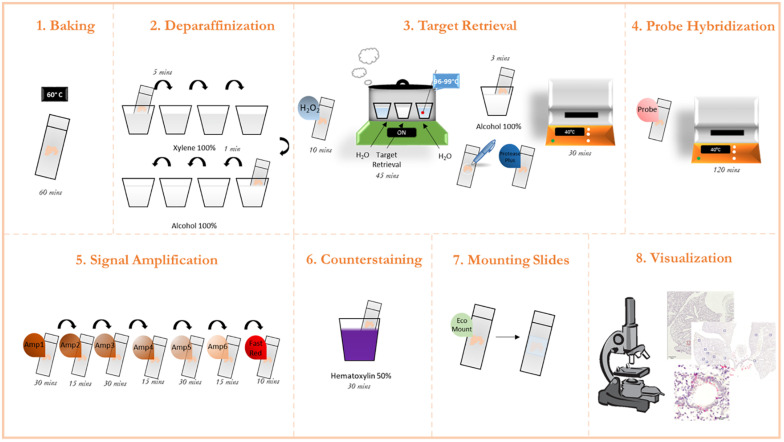
Graphic protocol scheme Summary of the RNAScope staining protocol and the different steps involved. (1) Baking of the paraffinized slides at 60°C. (2) Deparaffinization of slides with subsequent washes in xylene and ethanol. (3) Permeabilization of the slide and incubation with a target retrieval solution. (4) Probe hybridization with target RNA. (5) Signal amplification with subsequent staining with FastRed dye. (6) Counterstaining of the lung tissue with Hematoxylin. (7) Mounting of the slides. (8) Visualization under a brightfield microscope and image acquisition. (microscope schematic from https://favpng.com/

This methodology was adapted from the protocol guidelines provided by the ACD RNAScope platform (ACD, 2021). Furthermore, the company provides step-by-step short videos on each step of the process under “training videos” tab. A graphical overview of the process is presented in Figure 1.

### Animal Husbandry

Germ free animals with C57BL/6J background were generated and maintained by the Clean Mouse Facility of Department of Biomedical Research of the University of Bern kindly provided by Dr. Mercedes Gomez and Prof Sigfried Hapfelmeier. They were born and raised in flexible film isolators in at the University of Bern, transferred aseptically into sterile IVC cages provided with sterile food and water *ad libitum*. Seven to eight weeks colonized C57BL/6J mice were housed in a specific pathogen-free and climate-controlled animal facility at the University of Geneva. Mice were provided with water and pelleted diet *ad libitum*. All cages were provided with environmental enrichment in the form of nesting material and mouse houses. Procedures and experimentation were carried out in accordance with federal regulations of the Bundesamt für Lebensmittelsicherheit und Veterenärwesen (BLV) Switzerland (Tierschutzgesetz) and approved by an institutional review board and the cantonal authorities. Animal license GE/159/17.

### Extraction and Fixation of Mouse Lung Tissue

1.1 Euthanize animals (here done by controlled CO_2_ exposure). Confirm death by controlling from absence of reflex after pinching the footpad of the hind limbs.
1.2 Place the animal on the back and attach it with needles to a preparation board.
1.3 Wet the fur of the chest and abdomen with 70% ethanol. Avoid spilling ethanol in mouth or nose of the animal.
1.4 Lift the fur at the level of the lower tip of the sternum and make a horizontal incision.
1.5 Remove the fur from the mid abdomen to the neck by extending the cut along the lower end of the rib cage, followed by two lateral upward cuts toward the front limbs.
1.6 Flush fur attached to the exposed chest muscle tissue with 70% ethanol or sterile isotonic saline solution.
1.7 Make a small horizontal incision below the sternum and extend to this cut along the lower end of the rib cage.
1.8 Carefully puncture the diaphragm to collapse the lungs and open the rib cage by two lateral cuts toward the front limbs.
1.9 Carefully remove the lungs from the chest cavity. Pay attention not to put too much pressure with the forceps to the lung to avoid tissue damage.
1.10 Disconnect the attached connective tissue the trachea and the heart with scissors.
1.11 Immerse lungs in prepared 50 ml tubes with in PBS with 4%v/v freshly prepared paraformaldehyde solution (see “Materials” section) and stored at room temperature for 24 h. Make sure that the organ is fully immersed in the fixative. This can be achieved e.g., by adding the lid of a 15 ml tube upside down into the 50 ml tube, to hold the lung below the air-liquid interface. Of note, inflation of the lung with fixative is not recommended, since it could flush commensal bacteria out of their natural niche and might lead to artificial location in the organ.

### Paraffin Embedding

**FIGURE 2:**
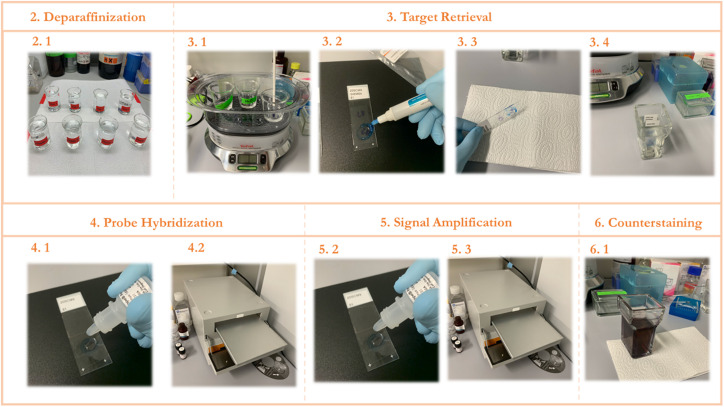
STEP-BY-STEP Visual protocol scheme. (1) Visual summary of the RNAScope staining protocol and the different steps involved. (2) Deparaffinization of slides with subsequent washes in xylene and ethanol. (3) Permeabilization of the slide and incubation with a target retrieval solution and subsequent hydrophobic barrier and washing. (4) Probe hybridization with target RNA. (5) Signal amplification with subsequent staining with FastRed dye. (6) Counterstaining of the lung tissue with Hematoxylin.

2.1 After fixation place lungs in cassettes, orient them properly according to the envisioned cutting program and dehydrate as follows.
2.2 Immerse lungs fully in 70% ethanol for 2 h at RT.
2.3 After 2 h Immerse Lungs fully a second time in 70% ethanol for 2 h at RT.
2.4 Immerse lungs fully in 90% ethanol for 1 h at RT.
2.5 Immerse lungs fully in 95% ethanol for 1 h at RT.
2.6 Immerse lungs fully in 100% ethanol for three consecutive steps of 30 min.
2.7 Transfer lungs into Histo-SAV I (Mallinckrodt Baker) for 30 min.
2.8 Immerse lungs fully in Histo-SAV II (Mallinckrodt Baker) for 30 min.
2.9 Immerse lungs fully in Histo-SAV III (Mallinckrodt Baker) for 30 min.
2.10 Transfer lungs into paraffin I for 2 h.
2.11 Transfer lungs into paraffin II for 4 h.
2.12 After hardening, cut paraffin blocks into 1 μM frontal sections using a microtome (Leica RM2235 Rotary Microtome).
2.13 Perform five consecutive transversal cuts at four different depths (1, 51, 101, and 151 μm). Cutting could be adapted according to experimental goals.
2.14 Transfer two consecutive slices of each depth onto one microscope slide and leave them to dry overnight at RT. Slides can be left either unstained or stained e.g., with Giemsa.
2.15 Submerge the slides in 20% Giemsa dye (Mallinckrodt Baker) diluted in distilled water.
2.16 Quickly dip the slides in 0.2% acetic acid (Sigma Aldrich) diluted in distilled water (organ samples should turn pink).
2.17 Quickly dip the slides in ethanol 90% (organ samples should turn blue).
2.18 Submerge the slides in isopropanol (Fluka) for 2 min.
2.19 Quickly dip the slides in ethanol 100%.
2.20 Quickly dip the slides in Histo-Sav (Mallinckrodt Baker).
2.21 Mount the slides following the protocol provided by UltraKit (Mallinckrodt Baker).
2.22 Place the slides containing the organ cuts in a dry oven for 1 h at 60°C to dry.
2.23 Place eight 50-mL beakers under a fume-hood and filled with either histological grade xylene or ethanol 99.9 grade (four beakers for each solution).x
2.24 Dip the dried microscope slides first into each xylene-containing beaker for 5 min each.
2.25 Continue with the ethanol-containing beakers for 1 min each and leave to air dry. Deparaffinized slides were used for the RNAScope procedure described in the next section (see also Figures 2–2.1).

### *In situ* Hybridization

3.1 Target Retrieval
  3.1.1 Lay the slides in the HybEZ tray and add enough drops of RNAscope Hydrogen Peroxide to cover the samples on each slide.
  3.1.2 Incubate at room temperature for 10 min.
  3.1.3 Tap the slides on top of absorbent paper to remove excess liquid and immediately submerge the slides in a reservoir containing distilled water.
  3.1.4 Lift the slides up and down for a proper wash.
  3.1.5 Repeat the washing step.
  3.1.6 Place two reservoirs containing RNAscope 1X Target Retrieval Reagent and Distilled water in a steam cooker (Tefal, #IP095536) machine set at 99°C.
  3.1.7 Add slides to the distilled water container for 10 seconds and move them into the container with RNAscope 1X Target Retrieval Reagent.
  3.1.8 Monitor the temperature with a thermometer.
  3.1.9 Cover the steam cooker and incubate for 45 min (refer to Figures 2–3.1). *This incubation time is susceptible to changes according to the samples*.
  3.1.10 Meanwhile, pre-warm HybEZ Oven at 40°C for 10 min.
  3.1.11 Remove slides from steam cooker and wash with distilled water.
  3.1.12 Move the slides to a container with Ethanol 99.9 grade for 3 min.
  3.1.13 Dry the slides in a dry oven at 60°C.
  3.1.14 Using the hydrophobic pen, draw a circular barrier around the samples on each slide and let it dry completely at room temperature for 5 min (refer to Figures 2–3.2).
  3.1.15 Lay the slides in the HybEZ tray and add enough drops of RNAscope Protease Plus to cover the samples on each slide.
  3.1.16 Incubate at 40°C inside the tray of the HybEZ Oven for 30 min. *This incubation time is susceptible to changes according to the samples*.
  3.1.17 Tap the slides on top of absorbent paper to remove excess liquid and immediately submerge the slides in a reservoir containing distilled water (refer to Figures 2–3.3, 2–3.4).
  3.1.18 Lift the slides up and down for a proper wash.
  3.1.19 Lay the slides in the HybEZ tray and add enough drops of Lysozyme (40 μg/mL) to cover the samples on each slide (optional).
  3.1.20 Incubate at 40°C inside the tray of the HybEZ Oven for 90 min (optional).
  3.1.21 Tap the slides on top of absorbent paper to remove excess liquid and immediately submerge the slides in a reservoir containing distilled water.
  3.1.22 Lift the slides up and down for a proper wash.

### Probe Hybridization

3.2.1 Pre-warm the HybEZ Oven at 40°C and the RNAScope probes at 37°C for 10 min.
3.2.2 Tap the slides on top of absorbent paper to remove excess liquid and lay them in the HybEZ tray.
3.2.3 Add enough drops of the appropriate probe to cover the samples on each slide (refer to Figures 2–4.1).
3.2.4 Incubate at 40°C inside the tray of the HybEZ Oven for 2 h (refer to Figures 2–4.2).
3.2.5 One at a time, tap the slides on top of absorbent paper to remove excess liquid and place it back on the tray.
3.2.6 Submerge the slides in a container filled with 1X Wash Buffer and lift them up and down for a proper wash.
3.2.7 Repeat the washing step.
3.2.8 Optional Step: Place the slides in a container with 5X SSC Buffer and leave them overnight.
3.2.9 Remove excess liquid by tapping the slide on top of absorbent paper and place them back on the tray.

### Signal Amplification

The next steps consist on the amplification of the probe signal by adding AMP1-4 reagents and incubating them at 40°C inside the tray of HybEZ Oven at 40°C.

Note: Always add enough drops of each reagent to cover the samples on the slides and remove excess liquid after incubation.

3.3.1 Add AMP 1 Reagent and incubate for 30 min (refer to Figures 2–5.1, 2–5.2).
3.3.2 Wash the slides twice with 1X Wash buffer.
3.3.3 Add AMP 2 Reagent and incubate for 15 min.
3.3.4 Wash the slides twice with 1X Wash buffer.
3.3.5 Add AMP 3 Reagent and incubate for 30 min.
3.3.6 Wash the slides twice with 1X Wash buffer.
3.3.7 Add AMP 4 Reagent and incubate for 15 min.
3.3.8 Wash the slides twice with 1X Wash buffer.

The next steps consist on the final amplification of the probe signal by adding AMP5-6 reagents and incubating them at room temperature.

3.3.9 Add AMP 5 Reagent and incubate for 30 min. *This incubation time can be modulated to adjust probe signal intensity*.
3.3.10 Wash the slides twice with 1X Wash buffer.
3.3.11 Add AMP 6 Reagent and incubate for 15 min.
3.3.12 Wash the slides twice with 1X Wash buffer.
3.3.13 Meanwhile, prepare a solution of Fast RED (A + B) by adding: 1 volume of Fast RED-B and 60 volumes of Fast RED-A.
3.3.14 Pipette enough volume of Fast RED mix to cover the samples in each slide (∼300 μL per slide) and incubate for 10 min at room temperature.
3.3.15 Tap the slides on top of absorbent paper to remove excess liquid and lay them in the HybEZ tray.
3.3.16 Rinse the slides with distilled water.

### Counterstaining

4.1 Submerge the slides in a container filled with Meyer’s solution diluted 1:2 for 2 min at room temperature (refer to Figures 2–6.1) *This dilution can be modulated to adjust staining intensity*.
4.2 Wash the slides with distilled water until the water is clear and has no traces of Meyer’s solution.
4.3 Submerge the slides in 0.02% ammonia diluted in distilled water and move the slides up and down a few times until the sample turns blue.
4.4 Wash the slides with distilled water.
4.5 Dry the slides at 60°C for at least 15 min or air dry until all visible liquid is evaporated.
4.6 Briefly dip the slides into a 50-mL beaker containing fresh histological grade xylene and add 1–2 drops of EcoMount (Vectamount) while the slides are still wet.
4.7 Cover the sample with a coverslip carefully to prevent formation of air bubbles and let it air-dry.

### Visualization

A large variety of imaging systems can be used for visualization. The following steps are thus solely to indicate a technical guidance and should be adapted to the existing infrastructure in each laboratory.

5.1 Visualize slides using a Olympus VS120 brightfield microscope (Olympus) and QuPath-0.2.1 Software.
5.2 Screen slides first with a 2× magnification for a quick visual scan of the overall distribution of positive signal (red-pink staining).
5.3 Choose a one random field from each slice and scanned again with the 100×/1.4 Oil objective using a multifocal approach. The VS-ASW creates a focus map with multiple coordinates defined by the user. This allows the optimal Z-position to be determined and saved on various parts of the sample, allowing a height profile of the sample to be compiled before detailed scan acquisition.
5.4 Process and analyze an average of 5–8 fields per sample at higher resolution. *This value can be adapted to the experimental requirements of each user*.
5.5 Determine positive staining of red-pink punctate dots around epithelial cells of airway ducts.

## Results

**FIGURE 3:**
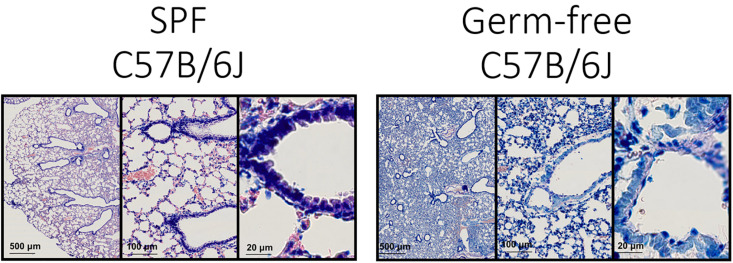
Lung architecture with Giemsa staining—Sections obtained by performing transversal cuts of whole lung and visualized under a brightfield microscope with a 100×/1.4 Oil objective.

In order to get an impression of the quality of tissue slices we performed Giemsa staining (Figure 3).

Technically this should allow the detection of bacteria in tissue (Tian et al., 2011; Morris et al., 2019), however, the density of flora and the nature of the bacteria could be limiting this staining technique.

Generally, the consultation of experienced histo-pathologists is advised in order to evaluate the quality of tissue slices.

**FIGURE 4:**
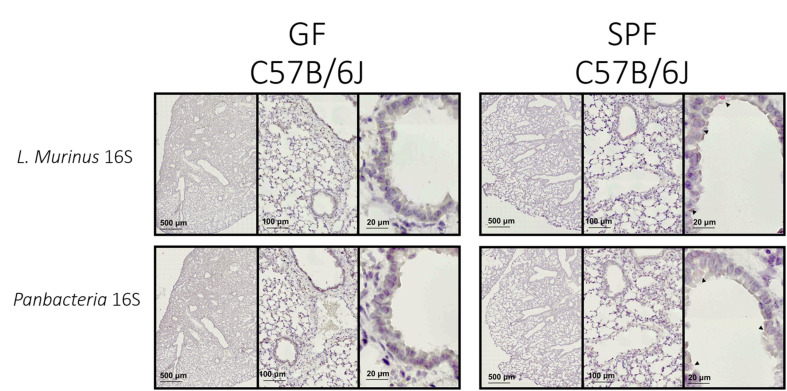
*L. murinus* and panbacteria visualization in lung sections of SPF and GF mice—Sections obtained by performing transversal cuts of whole lung and visualized under a brightfield microscope with a 100×/1.4 Oil objective. Black arrows indicate the presence of target RNA: *L. murinus* (top panel) or Panbacteria (lower panel).

The specific colorimetric staining for Panbacteria or *L. murinus* used here will provide a bright red-pink coloration of bacterial clusters (black arrows) attached to the medium or large airways (Figure 4–right panel). When adapted to other tissues, the density and location of commensal bacteria could differ (Welch et al., 2017). We recommend staining of consecutive slides with two probes (here Panbacteria and species specific) to independently confirm the presence of commensals. As a negative control we used lung tissue from axenic mice (Figure 4–left panel), which should remain free of staining. Alternatively, gnotobiotic animals lacking a certain species of bacteria could also be used as a negative control for the species-specific staining.

Conventionally housed mice or «wild» mice derived tissues would probably show a different distribution and colonization density with commensal bacteria. From previous experiments we know that the lung of SPF housed mice (in our facility) contains about 10^5^ CFU of bacteria when plated in rich chocolate agar or Columbia agar (Yildiz et al., 2020), most of which are *L. murinus* based on NGS data. 16S rRNA specific qPCR for this commensal revealed approximately 10^7^ genome equivalents. The discrepancy could be based on dead bacteria. Regardless, these numbers could be used as proxy for minimum colonization density required for successful ISH approaches.

### Critical Steps and Troubleshooting

• It is important to always use freshly prepared histological grade xylene, ethanol 99.9 grade and Meyer’s solution.
• The efficiency of the staining process primarily depends on the target retrieval step, which allows the permeabilization of the tissue granting the probe access to the target.
• The temperature of the steam cooker is crucial to be maintained constant (between 96 and 99°C) throughout the target retrieval incubation. The use of a thermometer on the steam cooker helps monitoring the temperature with the specified machine.
• We tried an additional stage of permeabilization since gram-positive bacteria such as *L. murinus* are known to have a thicker cell wall. For that we introduced a lysozyme incubation step for 90 min at 40°C. However, this additional exposure did not improve staining results (data not shown).
• Another important point during the staining process is the adjustment of the time of incubation during the signal amplification step. Amp5 incubation can be left for longer or shorter times for intensity regulation of the probe signal. On the same note, Meyer’s solution dilution can also be adjusted for a stronger counterstaining of the tissue slice. The fine-tuning of both these steps is helpful if the probe intensity is faint and contributes to a better contrast between counterstaining and target signal.

## Discussion

In this article we presented a general pipeline for ISH allowing visualization of commensal bacteria in an environment of low-colonization density. Combined with specific probes, detection of a given bacterial species in context of its natural organ context is possible. The technique provides a high signal to noise ratio as shown by the lack of background staining in axenic animals. In contrast to hybridization approaches targeting bacterial DNA (Choi et al., 2015), it further allows detection of mostly living bacteria since the targets are short lived bacterial RNAs. Similar approaches were already used to detect pathogenic bacteria in the densely colonized digestive tract of pigs (Jonach et al., 2014) or in human feces (Harmsen et al., 2002). As for metagenomic approaches the targeted bacterium does not require to be culturable. Nevertheless, FISH and ISH techniques are limited to a rather small number of targets, thus we propose this technique as complementary to genomic (Barfod et al., 2013; Pendleton et al., 2017) and culturomic approaches (Sibley et al., 2011; Browne et al., 2016).

We chose a colorimetric approach in order to better visualize the proximity to the host tissue (Yildiz et al., 2020), but in general fluorophore-labeled probes could also be used as shown in a recent study using pan-eubacteria specific fluorescent probes to detect lung commensal bacteria (Yun et al., 2014).

In a recently published study we estimated the total genome copy number of *L. murinus* in the lung of a SPF housed mouse as 10^7^, based on specific qPCR data (Yildiz et al., 2018). NGS data from the same study showed predominance of Lactobacilli in the lung (90–95%). In comparison to the fecal matter of ASF a systematic screen would be required to estimate the sensitivity of ISH for detection of very low abundant commensal bacteria. According to the above-mentioned relative abundance of Lactobacilli in the SPF house mouse lungs, we would expect a substantially reduced signal for the less abundant lung commensals (at least a factor of 10–20-fold reduction).

An interesting future expansion of this technique would be the combined detection of bacterial RNA and host RNA, to visualize, e.g., host responses of cells in the direct environment of commensal or pathogenic bacteria. Using distinct fluorescently labeled probes, such a staining is perfectly in accordance with the here proposed techniques and would require only little adaptation. In context of cancer diagnostics, a similar approach was used to confirm the presence of the bacteria strain *Acidovorax* as a potential biomarker for lung cancer (Greathouse et al., 2020). This approach would be complementary to dual RNAseq techniques, used to characterize interaction networks between bacteria and host from total organ homogenates (Westermann et al., 2016; Griesenauer et al., 2019).

A combination of several probes for the detection of multiple bacterial species in more complexly colonized animal would equally be of interest. For the intestinal tract FISH was already used to establish a biogeographical map indicating microbiota distribution on phylum level (Tropini et al., 2017; Welch et al., 2017). In mice with defined microbiota (Brugiroux et al., 2016), this approach would obviously be easier than in wild mice. Association of bacterial localization with metabolic function by metabolomic approaches (Marion et al., 2020) would be and additional, exciting approach to combine ISH for commensal bacteria with.

In combination with 16S specific rRNA gene NGS and metagenomic shot gun approaches we propose the use of complementing FISH or ISH approaches to localize commensal bacteria of interest in organ context.

## Data Availability Statement

The original contributions presented in the study are included in the article/supplementary material, further inquiries can be directed to the corresponding author/s.

## Ethics Statement

The animal study was reviewed and approved by the Direction de l’expérimentation animale, University of Geneva and SCAV Canton Geneva.

## Author Contributions

JPPB performed the ISH experiments and optimized the technique for detection of bacteria in lung tissue. Both authors wrote the article and approved the submitted version.

## Conflict of Interest

The authors declare that the research was conducted in the absence of any commercial or financial relationships that could be construed as a potential conflict of interest.
